# The Reaction of Aminonitriles with Aminothiols: A Way to Thiol-Containing Peptides and Nitrogen Heterocycles in the Primitive Earth Ocean

**DOI:** 10.3390/life8040047

**Published:** 2018-10-19

**Authors:** Ibrahim Shalayel, Seydou Coulibaly, Kieu Dung Ly, Anne Milet, Yannick Vallée

**Affiliations:** Univ. Grenoble Alpes, CNRS, Département de Chimie Moléculaire, Campus, F-38058 Grenoble, France; ibrahim.shalayel@univ-grenoble-alpes.fr (I.S.); bouahcoul@gmail.com (S.C.); lykieudung1005@yahoo.com.vn (K.D.L.); anne.milet@univ-grenoble-alpes.fr (A.M.)

**Keywords:** origin of life, prebiotic chemistry, thiol-rich peptides, cysteine, aminonitriles, imidazoles

## Abstract

The Strecker reaction of aldehydes with ammonia and hydrogen cyanide first leads to α-aminonitriles, which are then hydrolyzed to α-amino acids. However, before reacting with water, these aminonitriles can be trapped by aminothiols, such as cysteine or homocysteine, to give 5- or 6-membered ring heterocycles, which in turn are hydrolyzed to dipeptides. We propose that this two-step process enabled the formation of thiol-containing dipeptides in the primitive ocean. These small peptides are able to promote the formation of other peptide bonds and of heterocyclic molecules. Theoretical calculations support our experimental results. They predict that α-aminonitriles should be more reactive than other nitriles, and that imidazoles should be formed from transiently formed amidinonitriles. Overall, this set of reactions delineates a possible early stage of the development of organic chemistry, hence of life, on Earth dominated by nitriles and thiol-rich peptides (TRP).

## 1. Introduction

In ribosomes, peptide bonds are formed by the reaction of the amine group of an amino acid with an ester function. For non-ribosomal peptides, the amide formation involves the reaction of an amine on a thioester [1]. In both cases, mixed phosphoric carboxylic anhydrides are transiently formed. Esters, thioesters, and anhydrides are activated forms of the carboxylic acid function. Their intermediacy is mandatory and no significant C-N bond formation would occur directly from the reaction of an acid function with an amine [2]. What is true in today’s biology, was also true four billion years ago, when life was beginning its development in the terrestrial ocean. Activated derivatives had to be involved in the formation of prebiotic polymers. As a consequence, if acids were involved at some stage, a strong energy source was necessary. Nowadays, it is furnished by the cleavage of the triphosphate group of adenosine triphosphate [3].

**Figure 1:**
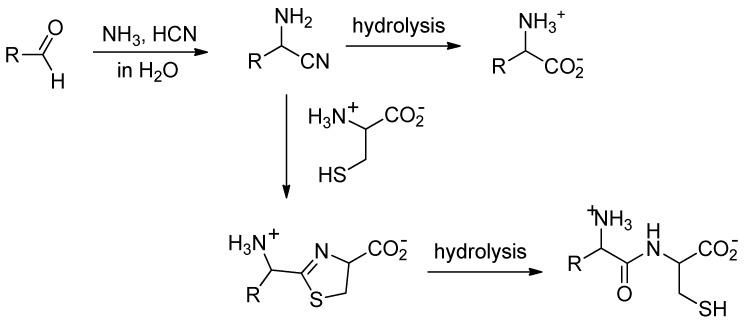
Strecker reaction followed by condensation of the obtained aminonitrile with cysteine.

Many simple aldehydes were probably present in the primitive ocean [4] and are plausible precursors for α-amino acids. Reacting with ammonia and hydrogen cyanide, they would have first given α-aminonitriles, which, upon hydrolysis, would have delivered amino acids (Figure 1) [5]. However, even though it is exothermic, the reaction of nitriles with water is a slow process [6]; so slow that, once formed in the ocean, aminonitriles would have had ample time to react with species more nucleophilic than water.

Nitriles are known to react with aminothiols to give thiazolines, which in turn can be hydrolyzed to mercaptoamides [7]. Starting from α-aminonitriles and cysteine, the expected products of this two-step process are dipeptides (Figure 1). In the early ocean, this could have been an efficient and selective process to thiol-containing dipeptides [8].

Compared to any activation process starting from acids, this nitrile scenario has the advantage of not necessitating any strong energy source. The acid does not need to be activated as it is delivered directly in an activated form by the Strecker reaction.

HCN has long been given an important role in prebiotic molecular evolution [9]. As it is largely distributed in space, having been observed in various regions, for instance, near carbon stars [10] and in a proto-planetary nebula [11], as well as in comets [12,13], it is highly possible that HCN was present on the early Earth. Furthermore, it has been postulated that it could have been formed when numerous asteroids struck our planet during the Late Heavy Bombardment [14]. It might have been produced photochemically in the atmosphere [15,16]. It was ejected from volcanoes [17] and submarine hydrothermal vents [18].

Hydrogen sulfide is another important small molecule in our hypothesis. It would have been necessary for the formation of cysteine. It has often been detected in space [19], *inter alia* in star forming regions [20], and in cold clouds [21], as well as in comets [22]. Furthermore, it is abundantly ejected from volcanoes [23,24], so there is no doubt that it was effectively present on the primitive Earth. Its presence permitted the synthesis of cysteine and homocysteine [25]. Homocysteine would have been obtained by a Strecker reaction starting from the addition of the product H_2_S onto acrolein (HSCH_2_CH_2_CHO). In a similar way, cysteine would have been synthesized from HSCH_2_CHO, itself possibly obtained from glycolaldehyde.

## 2. Experimental Section

Products (thiazolines, dipeptides…) were identified in reaction mixtures by NMR spectroscopy (^1^H and ^13^C) and mass spectrometry. No attempt at purifying them was made (except for **11** and **12**).

NMR monitored reactions were run in D_2_O solutions, in NMR tubes. NMR apparatus: Bruker Avance III 400 or 500. Classically, NMR experiments were run at concentrations of 5 × 10^−3^ to 5 × 10^−2^ mol/L.

For the mass experiment, H_2_O was used as the solvent. High-resolution mass spectra were recorded on a Waters G2-S Q-TOF mass spectrometer or on a LTQ Orbitrap XL (Thermo Scientific) spectrometer. Low resolution ESI analysis was performed on an Amazon speed (Brucker Daltonics) IonTrap spectrometer.

(*R*)-2-((*S*)-1-amino-3-(methylthio)propyl)-4,5-dihydrothiazole-4-carboxamide (**11**)

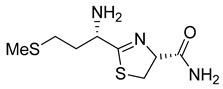



Met-CN (168 mg, 1.29 mmol) was dissolved in 15 mL H_2_O. Cys-NH_2_.TFA (280 mg; 1.29 mmol) was added. The pH of the solution was adjusted to 8 by adding Na_2_CO_3_. The solution was stirred at 45 °C for 2.5 h. The aqueous phase was extracted three times with ethyl acetate. The organic layer was dried over Na_2_SO_4_, filtered, and concentrated under vacuum. After purification by silica gel chromatography (1–10% MeOH/DCM), the thiazoline **11** was obtained as an orange oil (16% yield).

**HRMS** (ESI) for C_8_H_16_ON_3_S_2_: calc. *m*/*z* = 234.0735, Found *m*/*z* = 234.0740 [M + H]^+^. **^1^H-NMR** (D_2_O, 400 MHz) (δ, ppm): 5.08 (1H, t, *J* = 8.98 Hz, CH), 3.95 (1H, t, *J* = 6.57 Hz, CH), 3.65 (1H, t, *J* = 10.82 Hz, CH_2_), 3.46 (1H, dd, *J* = 11.30; 8.20, CH_2_), 2.56 (2H, t, *J* = 7.08 Hz, CH_2_), 2.07 (3H, s, CH_3_), 1.97 (2H, sep, *J* = 7.0, CH_2_). **^13^C-NMR** (D_2_O, 100 MHz) (δ, ppm): 182.99, 176.14, 77.00, 52.93, 34.89, 34.43, 29.18, 14.12.

(4*R*)-2-(1-amino-2-methylpropyl)-4,5-dihydrothiazole-4-carboxamide (**12**)

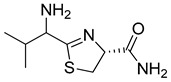



Val-CN.HCl (35 mg, 0.26 mmol) was dissolved in 5 mL H_2_O. Cys-NH_2_.TFA (57 mg; 0.26 mmol) was added. The solution was adjusted to pH = 7 by adding Na_2_CO_3_. The solution was stirred at 45 °C for 24 h. The aqueous phase was extracted three times with ethyl acetate. The organic layer was dried over Na_2_SO_4_, filtered, and concentrated under vacuum. After purification by silica gel chromatography (1–10% MeOH/DCM), the thiazoline **12** was obtained as a yellow oil (30% yield).

**HRMS** (ESI) for C_8_H_16_ON_3_S: calc. *m*/*z* = 202.1014, Found *m*/*z* = 202.1016 [M + H] ^+^. **^1^H-NMR** (D_2_O, 500 MHz) (δ, ppm): 4.40 (1H, m, CH), 3.75 (1H, dd, 5.68; 2.59 Hz, CH), 2.70–2.99 (2H, m, CH_2_), 2.12 (1H, sep, *J* = 6.60, CH), 0.85–0.94 (6H, m, CH_3_). **^13^C-NMR** (D_2_O, 125 MHz, both isomers were observed) (δ, ppm): 174.02–173.74, 169.70–169.43, 58.66–58.38, 55.65–55.63, 30.01–29.94, 25.30–25.02, 17.79–17.62, 16.85–16.65.

Theoretical calculations were carried out using the Gaussian09, Revision D.01 software. All the geometries were optimized using the B3LYP functional in conjunction with the 6-31g(d,p) basis set and the water solvent effects were described by using the polarizable continuum model (PCM), namely IEFPCM (integral equation formalism PCM) [26]. These optimizations were followed by a frequency calculation at the same level to ensure that the geometry was indeed a real minimum, i.e., all the second derivatives were positive.

## 3. Results

**Figure 2:**
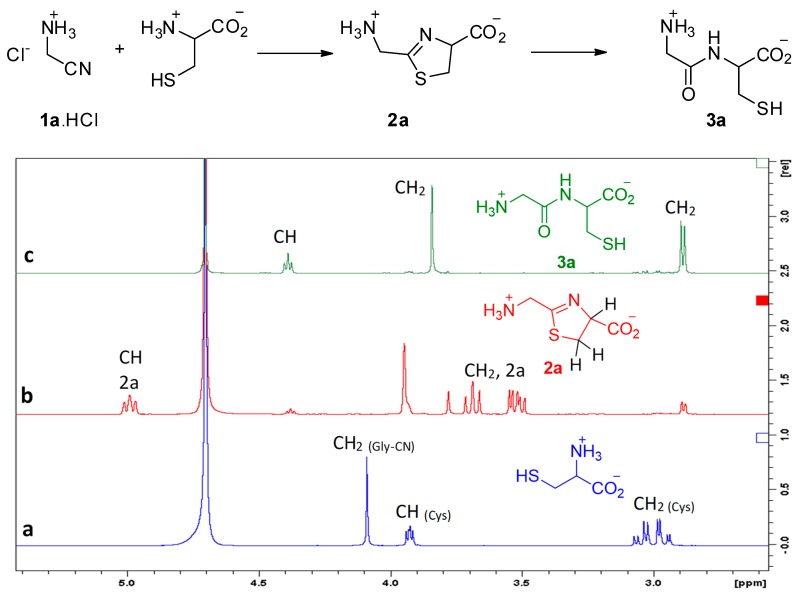
Reaction of aminoacetonitrile with cysteine, (**a**) mixture of starting materials, (**b**) mostly **2a**, (**c**) GlyCys **3a**. Conditions: room temperature, pH 6.5, concentration 10^−2^ mol/L.

We first studied the reaction of aminoacetonitrile (GlyCN **1a**, the nitrile derivative of glycine) with cysteine. Reactions were conducted in D_2_O solutions and followed by NMR spectroscopy. Representative ^1^H NMR spectra are shown in Figure 2. Formation of the expected thiazoline ring **2a** was evidenced by the apparition of signals at ca. 5 ppm (a triplet-like dd), and from 3.4 to 3.7 ppm (2 dd). After some time, new signals grew, including a triplet at 4.4 ppm and a thin doublet-like signal at ca. 2.9 ppm, both characteristic of Gly-Cys **3a**. We repeated this experiment many times, generally at a concentration of 5 × 10^−3^ to 5 × 10^−2^ mol/L, for practical NMR measurements. However, we also tested it at 3 10^−4^ mol/L, a concentration at which **2a** and **3a** were also obtained.

**Figure 3:**
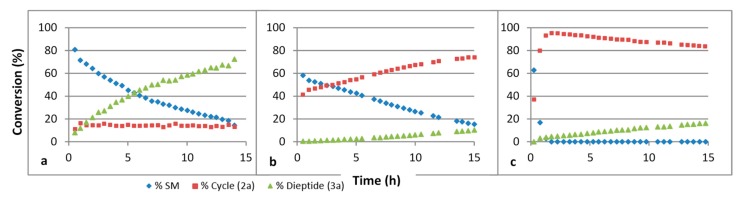
Evolution of a mixture of GlyCN and cysteine in D_2_O at 45 °C followed by ^1^H NMR, at various pH’s. (**a**) pH 4, (**b**) pH 6, (**c**) pH 8. SM: starting materials. Concentration 4 × 10^−2^ mol/L.

We have studied the influence of the pH on these reactions. The results are summarized in Figure 3. The ring formation is quicker under basic conditions. We believe that under such conditions, the thiol function is deprotonated, giving the more nucleophilic thiolate species. Under an acidic condition, the nucleophilic species is probably the thiol itself. The hydrolysis step is quicker under acidic conditions. This probably implies that the thiazoline ring is activated through protonation of the double bonded nitrogen atom before H_2_O addition.

We have also tested these reactions at 24 °C and 70 °C. Not surprisingly the process is quicker at a higher temperature, but also goes well at room temperature.

The conditions in the ocean four billion years ago are not precisely known. However, water was probably still hotter than now [27] and the presence of large amounts of CO_2_ in the atmosphere might imply that it was slightly acidic (nowadays, ocean’s pH is 8.1) [28]. Taking these considerations into account, we chose a temperature of 45 °C and a pH of ca. 5.5–6.5 as standard conditions.

**Figure 4:**
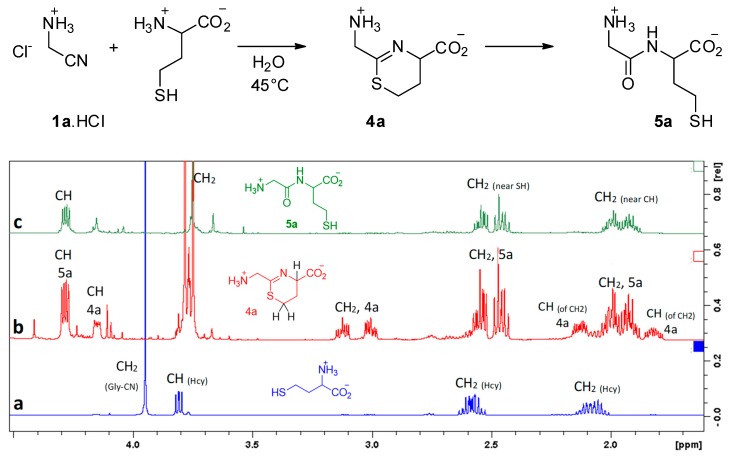
Reaction of homocysteine with GlyCN at 45 °C, pH = 6.5, 10^−2^ mol/L. ^1^H NMR’s show: (**a**) starting mixture, (**b**) reaction mixture after 6 h (**4a**/**5a** = 3/7), (**c**) after 24 h.

Under such conditions, we observed no reaction between aminoacetonitrile and any other proteinogenic amino acid that we tested (glycine, alanine, serine, methionine, aspartic acid, histidine, and lysine). It is worth noting that serine did not react. It appears that its alcohol function is not nucleophilic enough to attack the CN triple bond. Hence, the presence of a thiol function is mandatory. Indeed, homocysteine (Hcy) did react with a reaction rate similar to that observed with cysteine. In this case, the intermediate is the six-membered ring **4a**, and the final product is Gly-Hcy **5a** (Figure 4).

**Figure 5:**
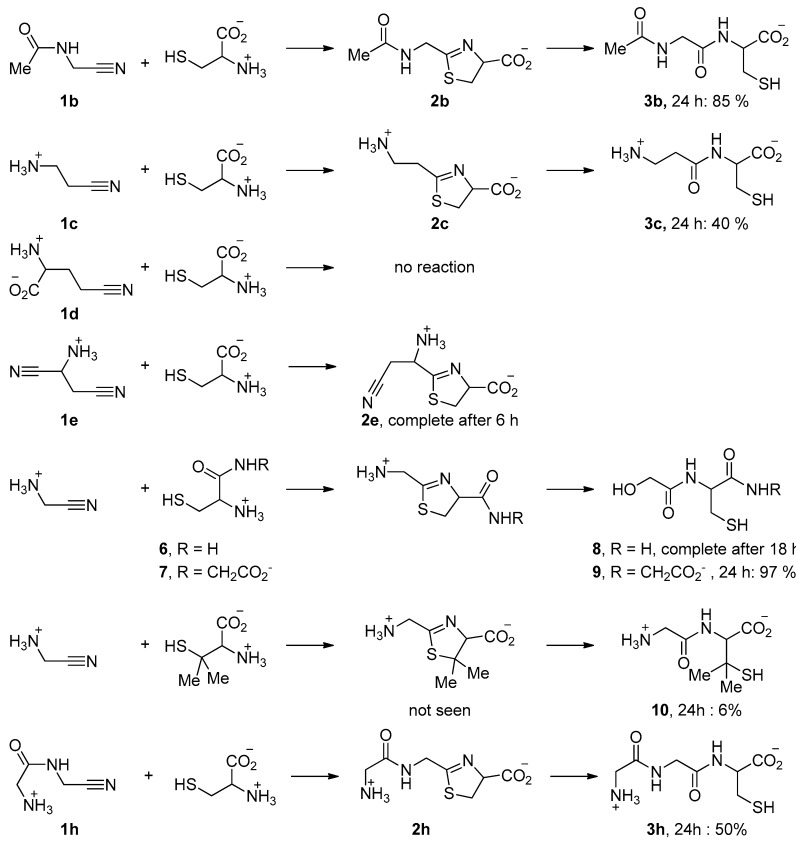
Some reactions of aminothiols with nitriles (45 °C, pH 5–6). The solvent was D_2_O. Reactions were monitored by ^1^H NMR.

**Figure 6:**
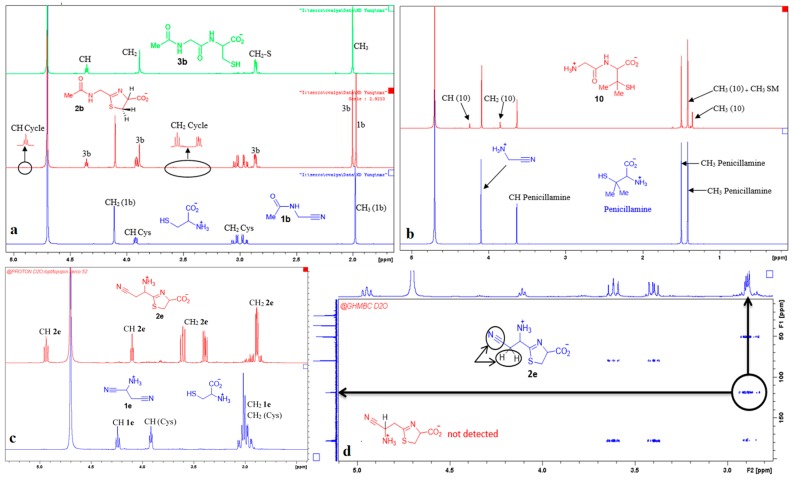
NMR spectra recorded during representative aminothiol + aminonitrile reactions. (**a**) Reaction of cysteine with *N*-acetyl aminoacetonitrile; (**b**) reaction of penicillamine with GlyCN; (**c**) reaction of aspartic acid bis-nitrile with cysteine; (**d**) 2D experiment demonstrating the regioselectivity of this last reaction towards α-nitrile.

**Figure 7:**
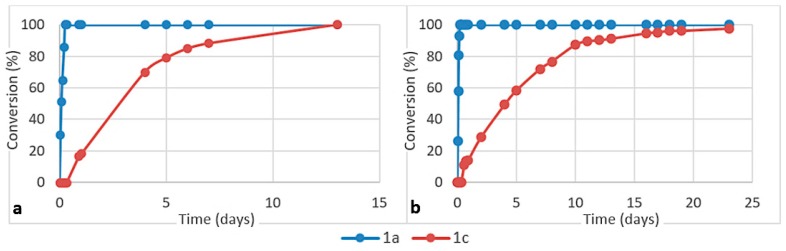
Consumption of aminoacetonitrile **1a** and β-aminopropionitrile **1c** in competition reactions with (**a**) cysteine and (**b**) homocysteine (ratio **1a**/**1c**/amino acid 1/1/2, pH ca. 6, 45 °C).

Some other representative results are presented in Figure 5. They show that the acid function of cysteine can be replaced by a primary or secondary amide. When Cys-Gly was used, the tripeptide Gly-Cys-Gly **9** was obtained with a very good conversion. *N*-Acetyl aminoacetonitrile **1b**, which can be considered as a model for any other *N*-acyl acetonitrile, including cyano-terminated peptides, also reacted with a good rate (Figure 6a). In contrast, the reaction was slower when aminoacetonitrile was replaced by β-aminopropionitrile **1c** (Figure 7). In these two last examples, the hydrolysis step was quick. No reaction was observed with the γ-nitrile of glutamic acid **1d** [29]. The selectivity in favor of α-aminonitriles was also exemplified when the bis-nitrile derived from aspartic acid **1e** [30] was used. In this case, only the α-aminonitrile reacted, giving the corresponding thiazoline **2e,** which was stable under these conditions (Figure 6c,d).

Finally, we also tested the reactivity of penicilamine, a sterically hindered aminothiol. In this case, the reaction was very slow, probably because of the bulkiness of the gem-dimethyl substituents. Furthermore, the only detected product was the final dipeptide **10** (Figure 6b). This might be due to the electron donating property of the methyl groups, making the nitrogen atom of the intermediate thiazoline ring more basic. Protonation of this nitrogen atom would thus be easier, hence the hydrolysis step quicker.

In order to explain the observed selectivity, we calculated the level of the π* orbital of a series of nitriles (Table 1).

The lowest calculated orbital was that of the protonated form of aminoacetonitrile. Such a level would explain its greater reactivity compared to other nitriles. Noticeably, the non-protonated form of aminoacetonitrile is predicted to be much less reactive and so is probably not involved in the reaction mechanism. Also, the π* orbital of β-propionitrile is higher (it is less reactive) and the simplest γ-aminonitrile is predicted to be even less reactive (no reaction from glutamic nitrile). In contrast, α-substitution of aminoacetonitrile, as in α-propionitrile, should not significantly alter its reactivity.

**Figure 8:**
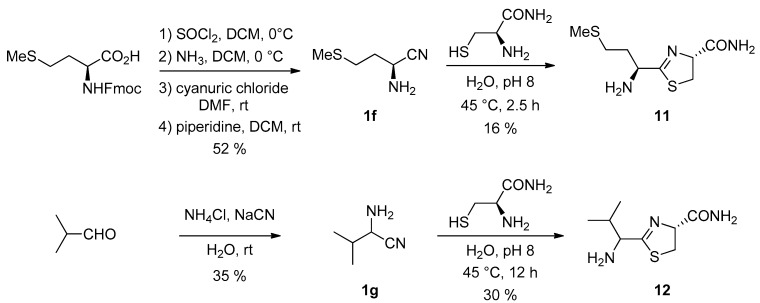
Synthesis of MeCN **1f** and ValCN **1g** and their reaction with cysteine amide.

We studied this substitution effect using the nitriles derived from two other amino acids (Figure 8). l-Methionine nitrile **1f** [31] was prepared from N-protected L-methionine in a three-step process. Valine nitrile **1g**, was prepared as a racemic mixture using a Strecker reaction from the corresponding aldehyde [32].

Their reaction with cysteine amide [33] was studied (Figure 8). In these cases, we were able to isolate the intermediate cycles in pure form (as a 1/1 mixture of diastereoisomers from racemic ValCN). The deceptive isolated yields, despite the slightly basic conditions we used, which should have slowed the hydrolysis step, were probably due to important hydrolysis during column chromatography on silica. In addition, we found that the rate of hydrolysis in water of the valine-derived thiazoline **12** was much slower than the one of the methionine derivative **11** (and of the simplest Glycine derivative **2a**). This is probably due to the presence of the bulky isopropyl group in **12**.

On the basis of our experiments, we propose that AA-Cys and AA-Hcy dipeptides were over-represented in the primitive ocean (compared to non-thiol-containing dipeptides).

**Figure 9:**
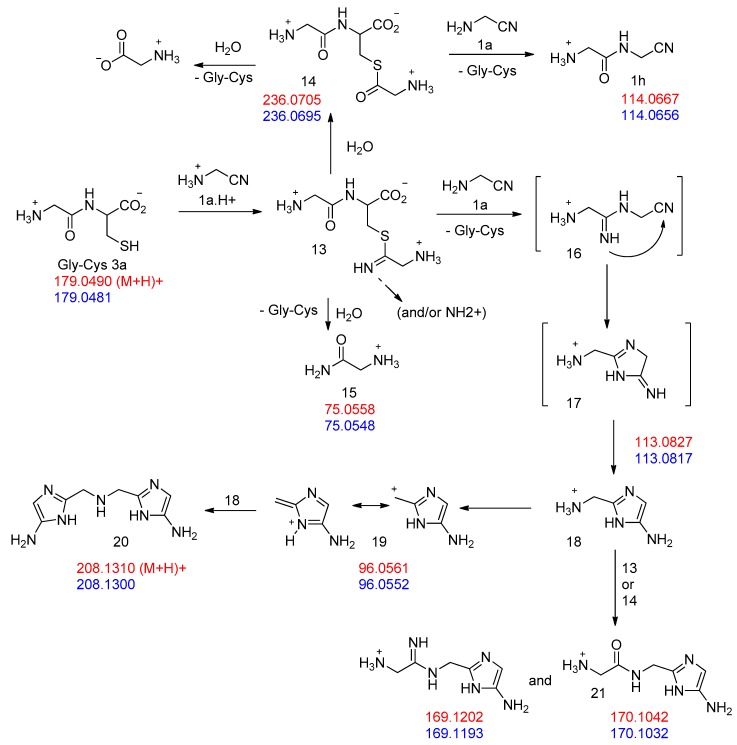
Proposed pathways in the reaction of excess GlyCN **1a** with GlyCys **3a**. Mass attribution (red, calculated; blue, found).

**Figure 10:**
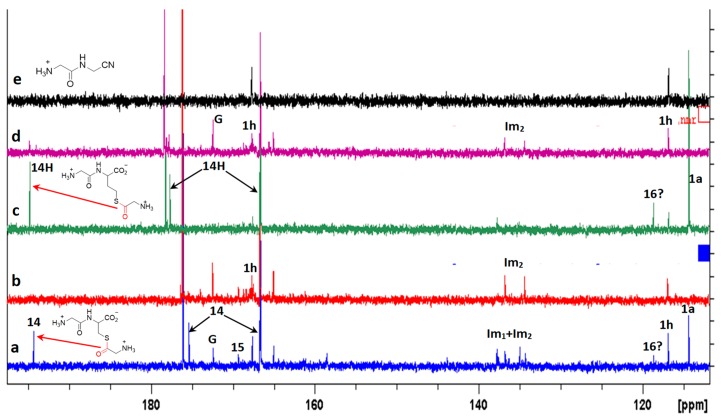
^13^C NMR spectra recorded during the reaction of an excess GlyCN **1a** with GlyCys **3a** (from **1a**. HCl + cysteine) or GlyHcy **5a** (from **1a**. HCl + homocysteine) at 45 °C, pH 6.5. (**a**) with **3a** 20 h after mixing **1a**. HCl and cysteine; (**b**) after 70h; (**c**) with **5a** 20 h after mixing **1a**. HCl and homocysteine; (**d**) after 70h; (**e**) reference spectrum of **1h**. **G**: glycine. Peaks corresponding to at least two products were detected near 135–140 ppm. They might correspond to two different imidazoles (named **Im_1_** and **Im_2_**). **14H**: the homocysteine thioester analogue of **14.** Big peaks at 166.65 (166.66) and 176.07 (178.33) belong to **3a** (and **5a**). One peak of both **14** and **14H** sticks to the foot of the 166.6 ppm peak of **3a** and **5a**.

These dipeptides are thiols and as such, could be major players in a “thioester world” [34]. Indeed, when we mixed Gly-Cys **3a** (obtained from a 1 to 1 mixture of GlyCN and cysteine) with an excess of GlyCN in D_2_O solution at 45 °C (Figure 9), a peak was observed at 194.16 ppm in the ^13^C NMR of the reaction mixture (Figure 10). Such a chemical shift is characteristic of the thioester function. We believe that it belongs to compound **14**. We also noticed the formation of glycine amide **15**. These products would both derive from the first formed C=N double-bonded addition product **13**. The thioester was partly hydrolyzed to give glycine, but we were also able to characterize, among the reaction products, the amidonitrile Gly-GlyCN **1h** (^13^C NMR: 27.58, 40.34, 116.74, 167.58 ppm), meaning that the thioester reacted with the non-protonated amino group of GlyCN (which is possible because of the low pKa of GlyCN: 5.55 [35]). For instance, in an experiment in which we used globally 4 eq. of GlyCN (relative to cysteine), after two days at 45 °C, the observed GlyOH/GlyNH_2_/GlyGlyCN ratio was found to be 21/37/42. This demonstrates that Gly-Cys is able to promote the formation of a peptide bond from a nitrile. Similar results were obtained for Gly-Hcy. In addition, as our theoretical calculations predicted (Table 1), when Gly-GlyCN **1h** was mixed with cysteine, Gly-Gly-Cys **3h** [36] was readily formed (Figure 5), demonstrating that not only dipeptides, but also tripeptides, could have been formed by this process in the primitive ocean.

**Figure 11:**
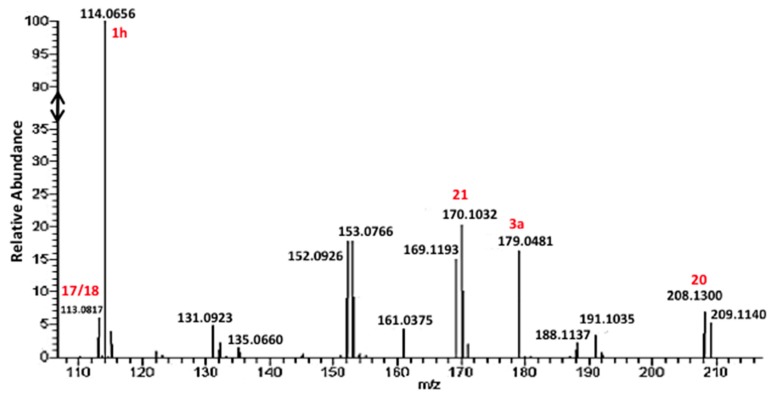
Mass spectrum of a reaction of an excess GlyCN **1a** with GlyCys.

However, the formation of other products was also evidenced in the reaction of cysteine with excess GlyCN. Thus, in the ^13^C NMR spectra, peaks at 130–140 ppm were observed (Figure 10). The mass spectrum of a reaction mixture in water also showed the formation of various products (Figure 11, see Supplementary Materials for complete spectrum and further mass attributions). This mass spectrum first confirmed the presence of Gly-GlyCN **1h** (protonated, found 114.0656, calcd 114.0667). Another mass was detected at 113.0817. It could be attributed to the amidine **16** (calcd 113.0827). However, in accordance with the observed ^13^C NMR of the mixture, we propose that this amidine cyclized and that this mass peak should be attributed to the imidazole **18**. Indeed, the cyclization of an amidinonitrile similar to **16** into an aminoimidazole (5-amino-2-methyl-1H-imidazole) has already been reported [37]. At least some of the other products observed in the mass spectrum would be evolution products of **18**. For instance, this imidazole could lose ammonia to give the stabilized cation **19** that would in turn react with **18** (in its free amine form) to yield the bis-imidazole **20** (M+H, found 208.1300, calcd 208.1310). **18** itself could react with thiolester **14** to give the amide **21**. Other structures are possible (see Supplementary Materials).

**Figure 12:**
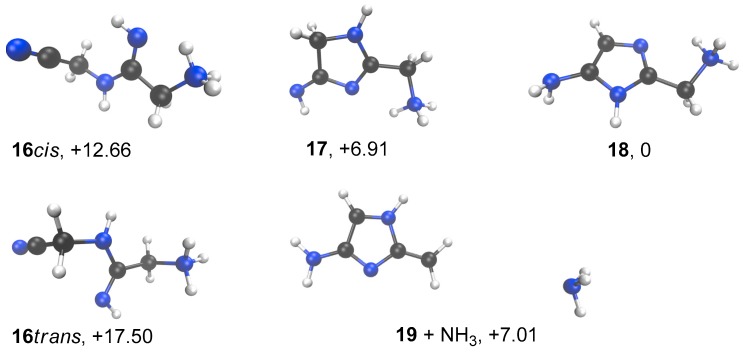
Minimized conformation for compounds **16** to **19**. B3LYP/6-31+G** with a continuum to mimic the solvent effect of water. ∆G’s relative to **18** in kcal/mol.

In order to further ascertain the cyclization of the imino-compound **16**, we calculated its stability in comparison to cyclized forms. We used the strategy described previously for Table 1 results, with the 6-31+G(d,p) basis set (Figure 12). Not surprisingly, **18** was calculated to have free enthalpy 12.7 kcal/mol lower than **16** and 6.9 kcal/mol more stable than **17**.

Interestingly, it was found that the dissociation of **18** into **19** + NH_3_ only costs around 7 kcal/mol. The ΔH of dissociation is nearly 19 kcal/mol (18.67 kcal/mol), but due to the dissociative character of the process, the ∆G value drops to 7.01 kcal/mol. This process does not show a well-defined TS. Thus, we think that the formation of cation **19** proposed in Figure 9 is a plausible event.

**Figure 13:**
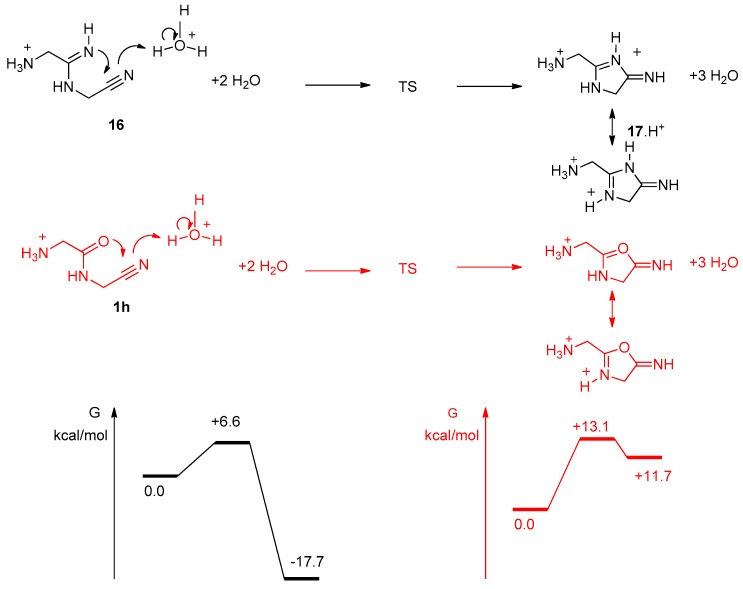
Reaction pathways for the cyclisation step from **16** (black), and **1h** (red). Level of calculation B3LYP/6-31+G**/SCRF(water).

We studied more precisely the cyclization step from **16** to **17** (Figure 13). H_3_O^+^ was used to promote the reaction and to give a proton to the nitrogen atom of the nitrile group, which becomes part of the exocyclic imine of **17**. Two explicit molecules of water were introduced, in addition to the water continuum. It appeared that the cyclization step should be exocyclic and quick, with a low level TS (activation energy of 6.6 kcal/mol). This is another confirmation that the compound of mass 113.0817 we observed was not **16**, but indeed the imidazole **18** (resulting from a simple proton migration from **17**). It is noticeable that similar calculations for the potential cyclization of the amide **1h** into an oxazole indicated that this reaction should not happen. Indeed, we never observed it experimentally. In sharp contrast with **16**, **1h** (GlyGlyCN) is stable.

## 4. Conclusions

A world containing small peptides and heterocycles, based on the chemistry of thiols and nitriles, can be delineated. It could have persisted as long as a significant amount of HCN was present in the ocean and permitted the synthesis of aminonitriles from aldehydes. In this “cyano-sulfidic” world [14], thiol-containing peptides would have been the most important molecules. We propose to name it the “Thiol Rich Peptide (TRP) world” [8]. In such a world, not only dipeptides, but also tripeptides, would have been formed. For instance, any dipeptide nitrile AA1-AA2CN (the simplest example being Gly-GlyCN) produced from the reaction of H_2_NAA2CN with a thioester of AA1, would react with cysteine to give the tripeptide AA1-AA2-Cys, and with homocysteine to give AA1-AA2-Hcy. Could some of these tripeptides have been the very first catalytic triades [38]? Indeed, we have demonstrated that even dipeptides like GlyCys (but not monomeric cysteine) are able to promote the formation of peptide bonds from nitriles. They are also able to promote the formation of imidazoles. Such heterocycles play an important role in today’s biochemistry. Of special interest is the simplest aminoimidazole, which, as its ribonucleotide derivative (AIR) [39], is an intermediate in the de-novo synthesis of inosine monophosphate (IMP), hence of purine nucleotides. Thus, imidazoles could have established a bridge from peptides to nucleic acids.

## Figures and Tables

**Table 1:** Calculated level of the π* orbital of various nitriles.

Nitrile		π* Value	Reaction Rate
H_3_N(+)CH_2_CN 1a protonated		−0.03632	quick
H_2_NCH_2_CN 1a		0.01698	No reaction?
H_3_N(+)CH(CH_3_)CN		−0.03010	quick
H_3_CCONHCH_2_CN 1b		0.00504	slower
H_3_N(+)CH_2_CONHCH_2_CN 1h		0.00353	quick
H_3_N(+)CH_2_CONHCH_2_CONHCH_2_CN		−0.00547	quick
H_3_N(+)CH_2_CH_2_CN 1c		0.00616	slower
H_3_N(+)CH_2_CH_2_CH_2_CN		0.01556	No reaction
H_3_CCN		0.03491	No reaction
Aspartic acid bis-nitrile 1e	αCN	−0.03566	Reacts at αCN
	βCN	−0.00500	

## Supplementary Materials

The following are available online at http://www.mdpi.com/2075-1729/8/4/47/s1. HRMS of **1a** + cysteine and + homocysteine reaction mixtures. NMR spectra of **11** and **12**. ESI mass spectrum and ^13^C NMR of a cysteine + excess GlyCN reaction mixture. Additional data for theoretical calculations.
