# Supplementary material for: The Reaction of Aminonitriles with Aminothiols: A Way to Thiol-Containing Peptides and Nitrogen Heterocycles in the Primitive Earth Ocean
Source: Life (Basel). 2018 Oct 19;8(4):47. doi: 10.3390/life8040047 (PMC6316830; doi:10.3390/life8040047)
Supplement: Supplementary file 1 [file life-08-00047-s001.pdf]

Supplementary material for:

The reaction of aminonitriles with aminothiols: a way to thiol containing peptides and nitrogen heterocycles in the primitive Earth ocean

Ibrahim Shalayel, Seydou Coulibaly, Ly Kieu Dung, Anne Milet, Yannick Vallée*

Univ. Grenoble Alpes, CNRS, Département de Chimie Moléculaire, Campus, Grenoble, France

1- HRMS of **2a**, **3a**, **4a** and **5a** 2

2- Copies of NMR spectra (**11** and **12**) 3

3- ESI analysis of a cysteine + excess **1a** reaction mixture 7

4- 13C NMR of a cysteine + excess **1a** reaction mixture 12

5- Theoretical calculation data for Figure 11 13

HRMS of reaction mixtures

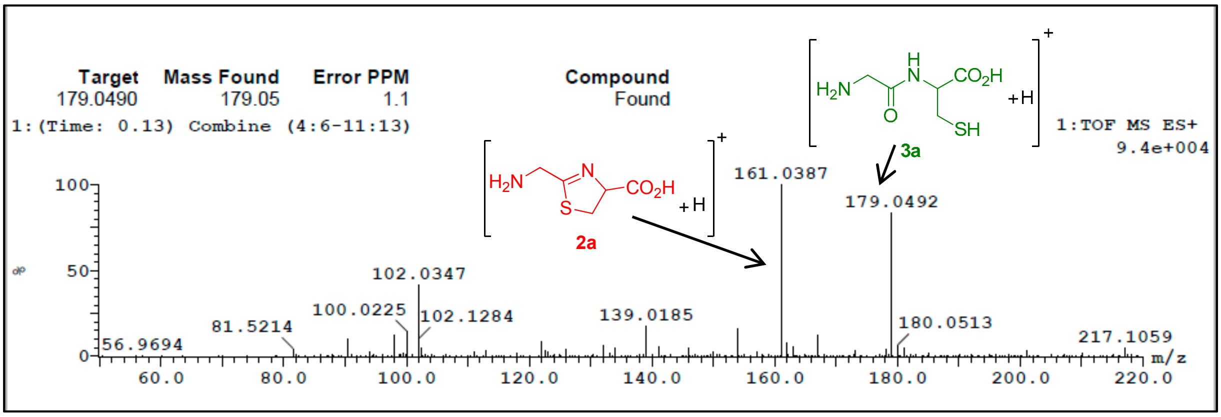

2a $[M+H]^+$ calcd 161.0385, found 161.0387

3a $[M+H]^+$ calcd 179.0490, found 179.0492

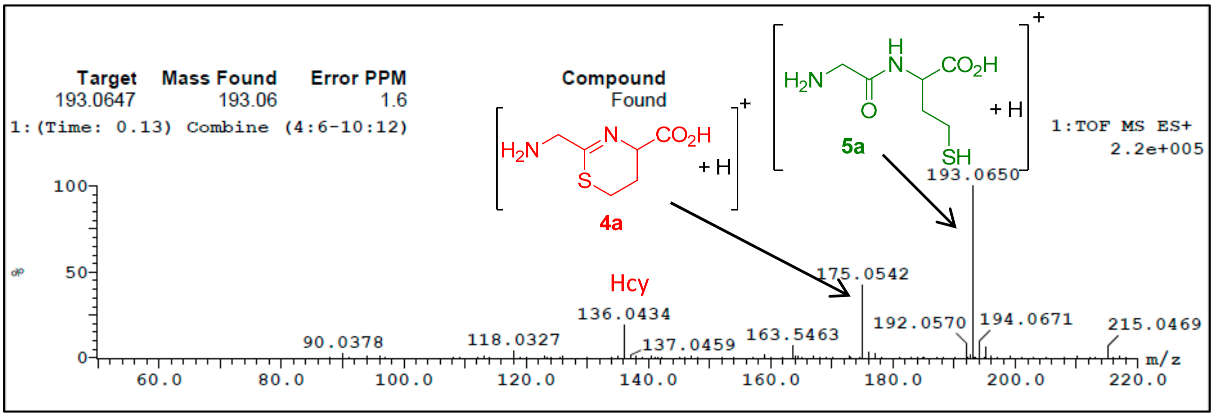

4a $[M+H]^+$ calcd 175.0541, found 175.0542

5a $[M+H]^+$ calcd 193.0647, found 193.0650

^{1}H NMR: L-Met-Cys-NH2 thiazoline **11**

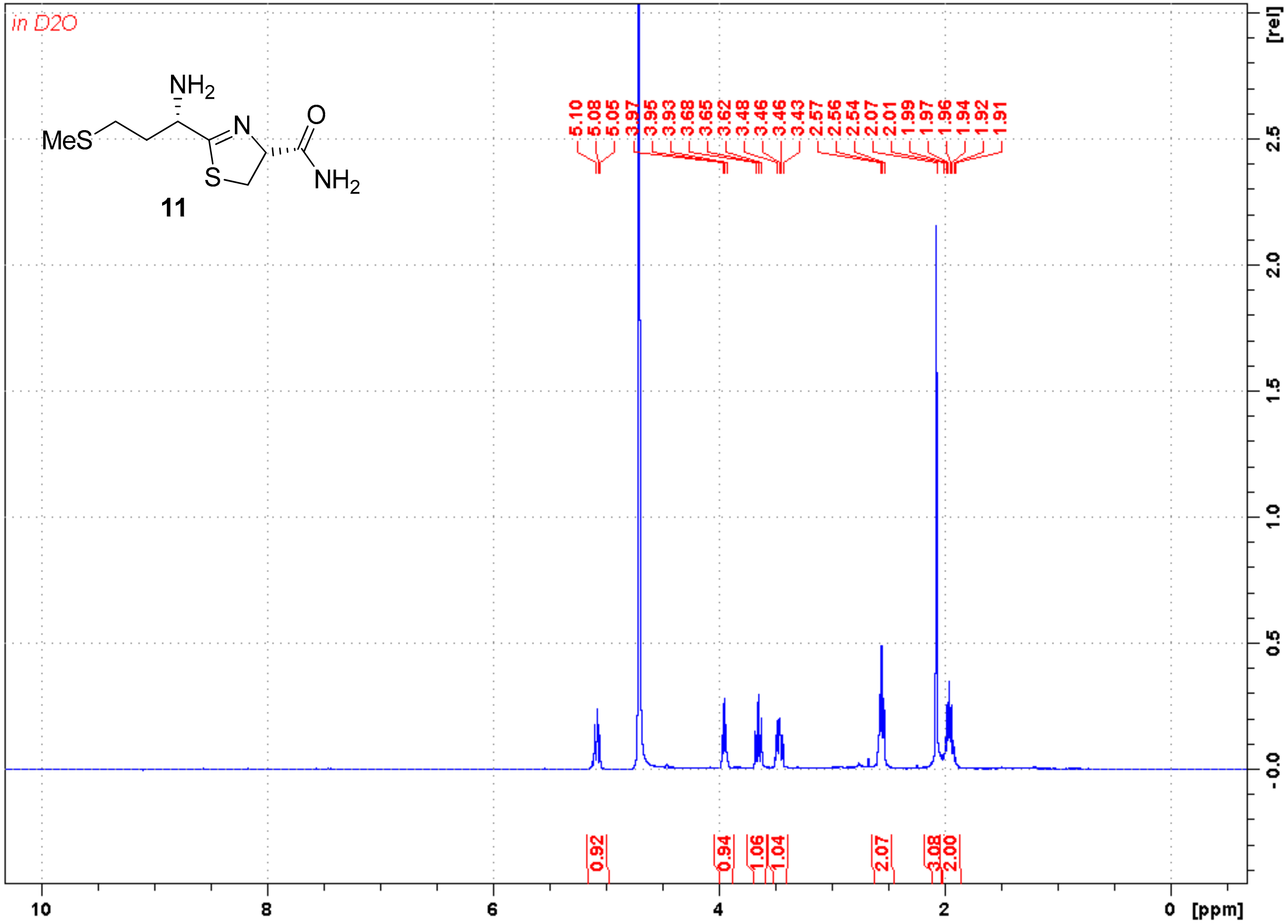

^{13}C NMR: L-Met-Cys-NH2 thiazoline **11**

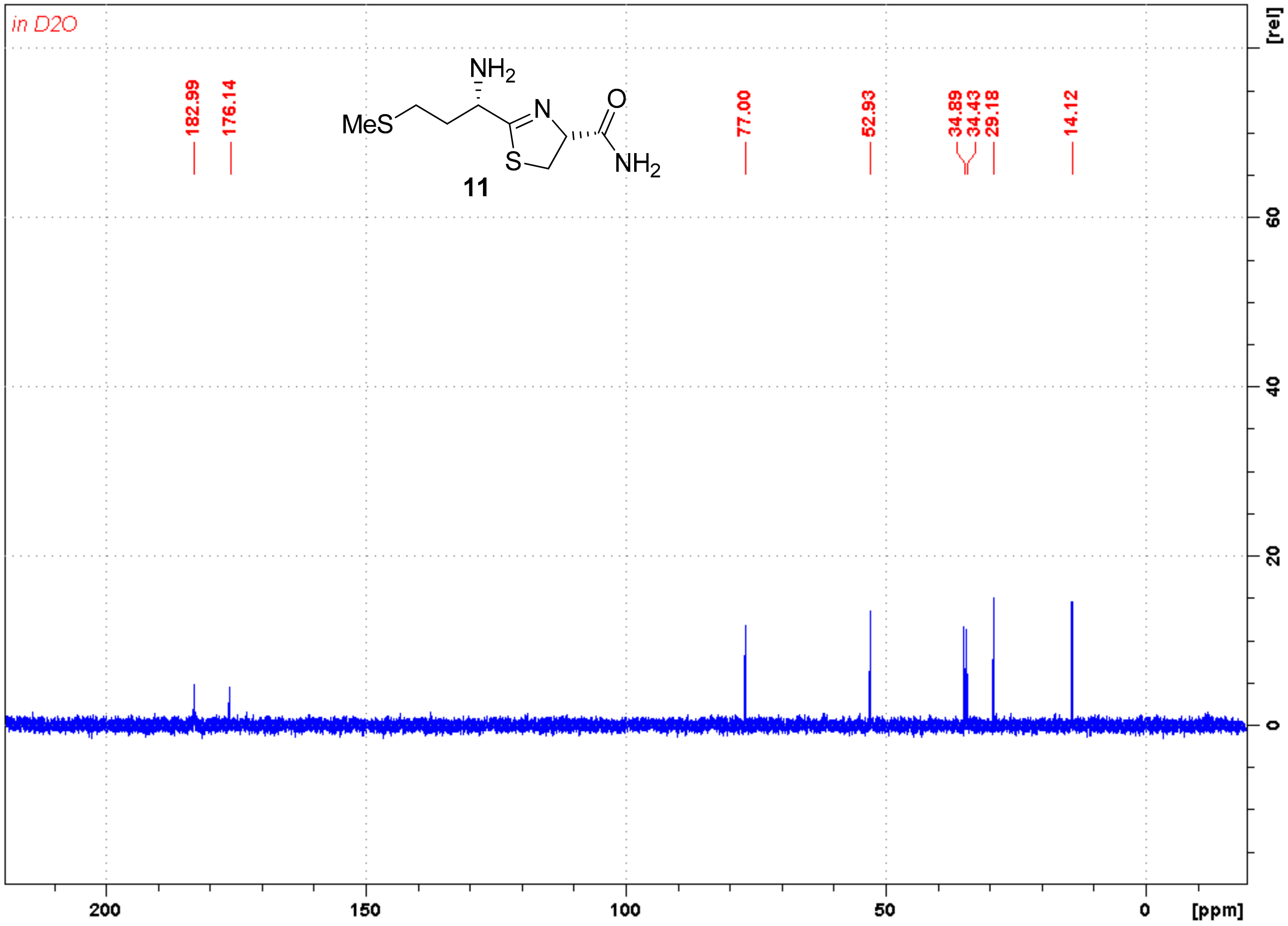

^{1}H NMR: Val-Cys-NH_2 thiazoline **12**

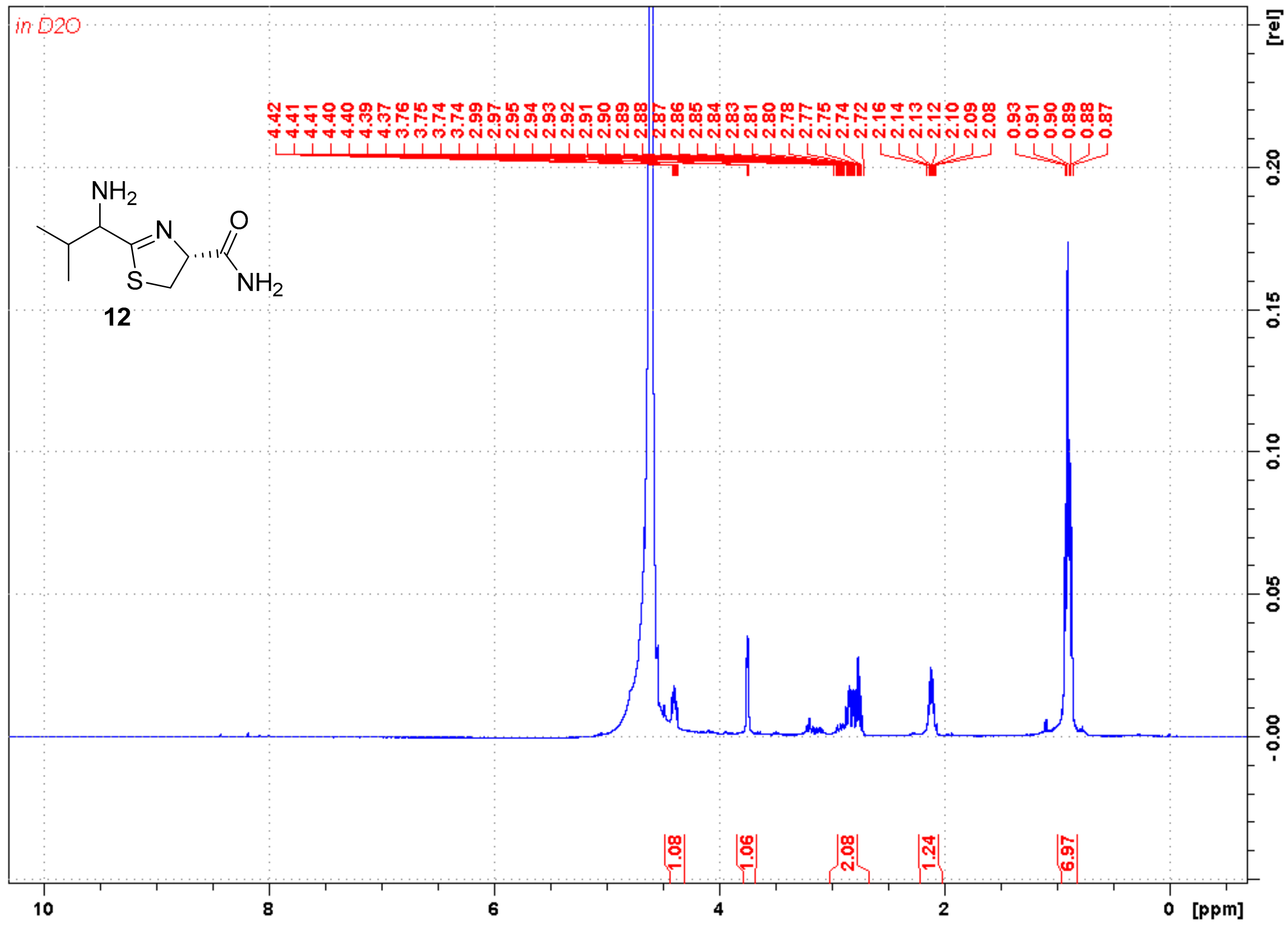

^{13}C NMR: Val-Cys-NH_2 thiazoline **12**

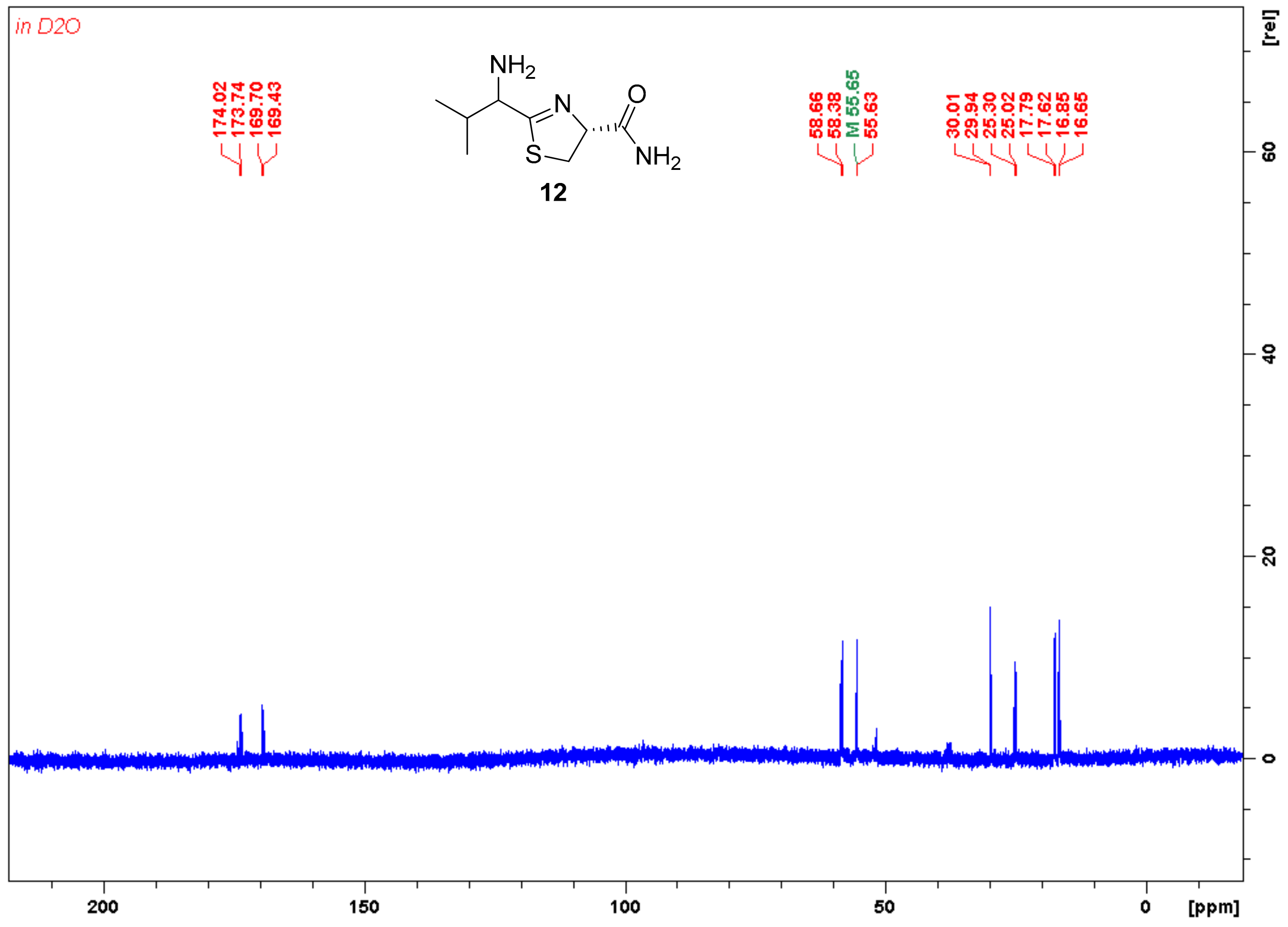

ESI spectrum of a cysteine + excess **1a** reaction mixture (1): ordinate scale x 1

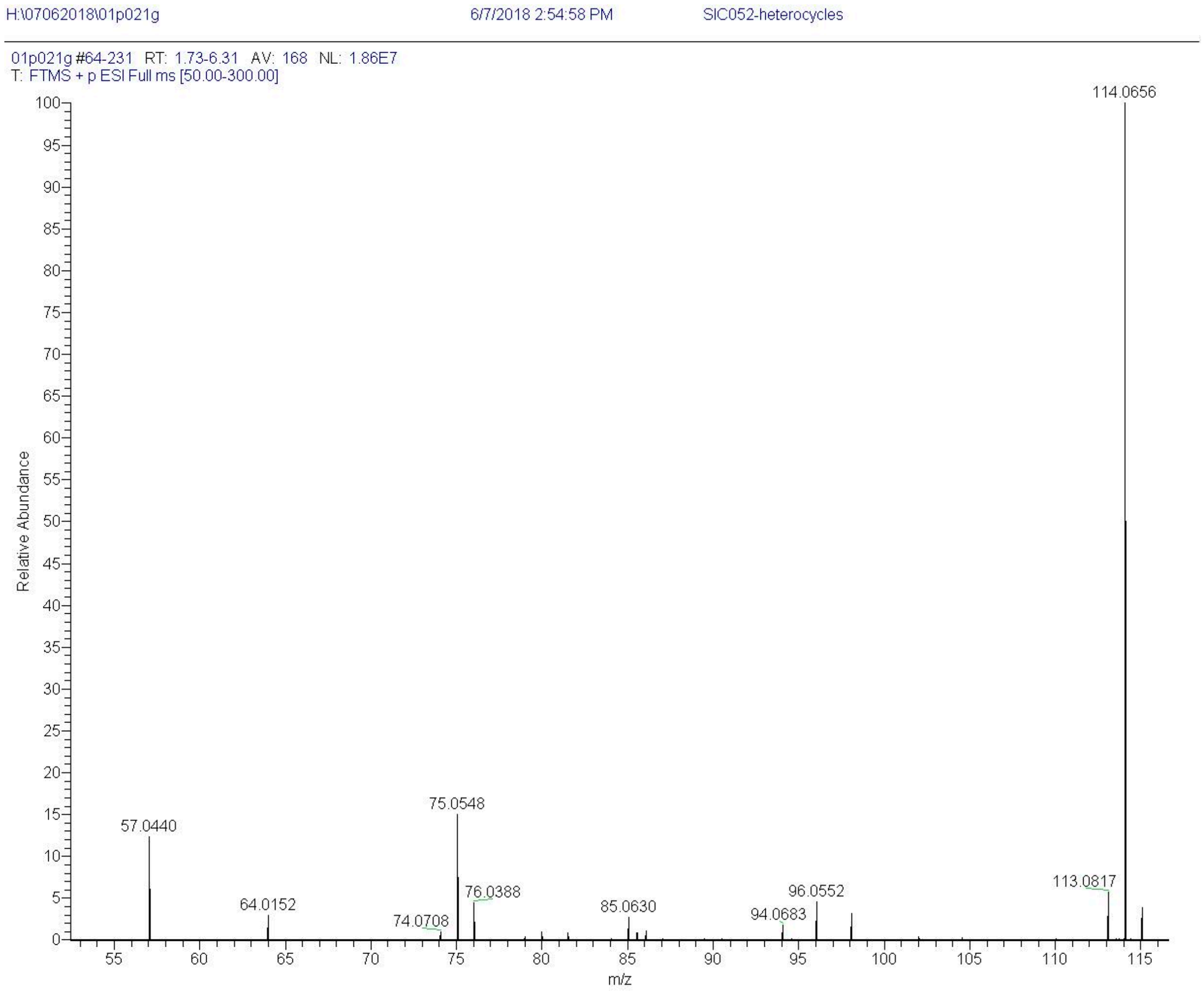

ESI spectrum of a cysteine + excess **1a** reaction mixture (2): ordinate scale x 6

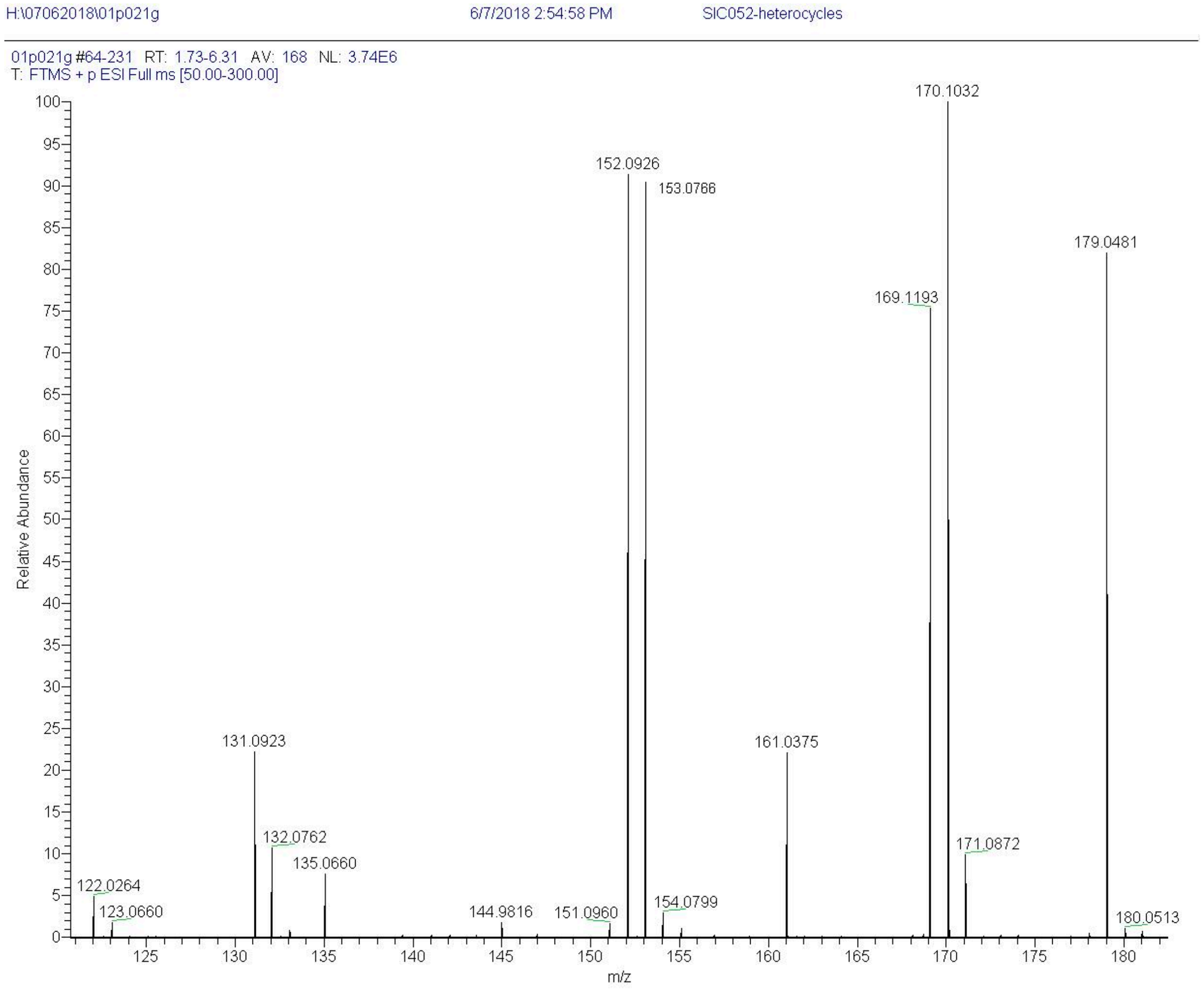

ESI spectrum of a cysteine + excess **1a** reaction mixture (3): ordinate scale x 12

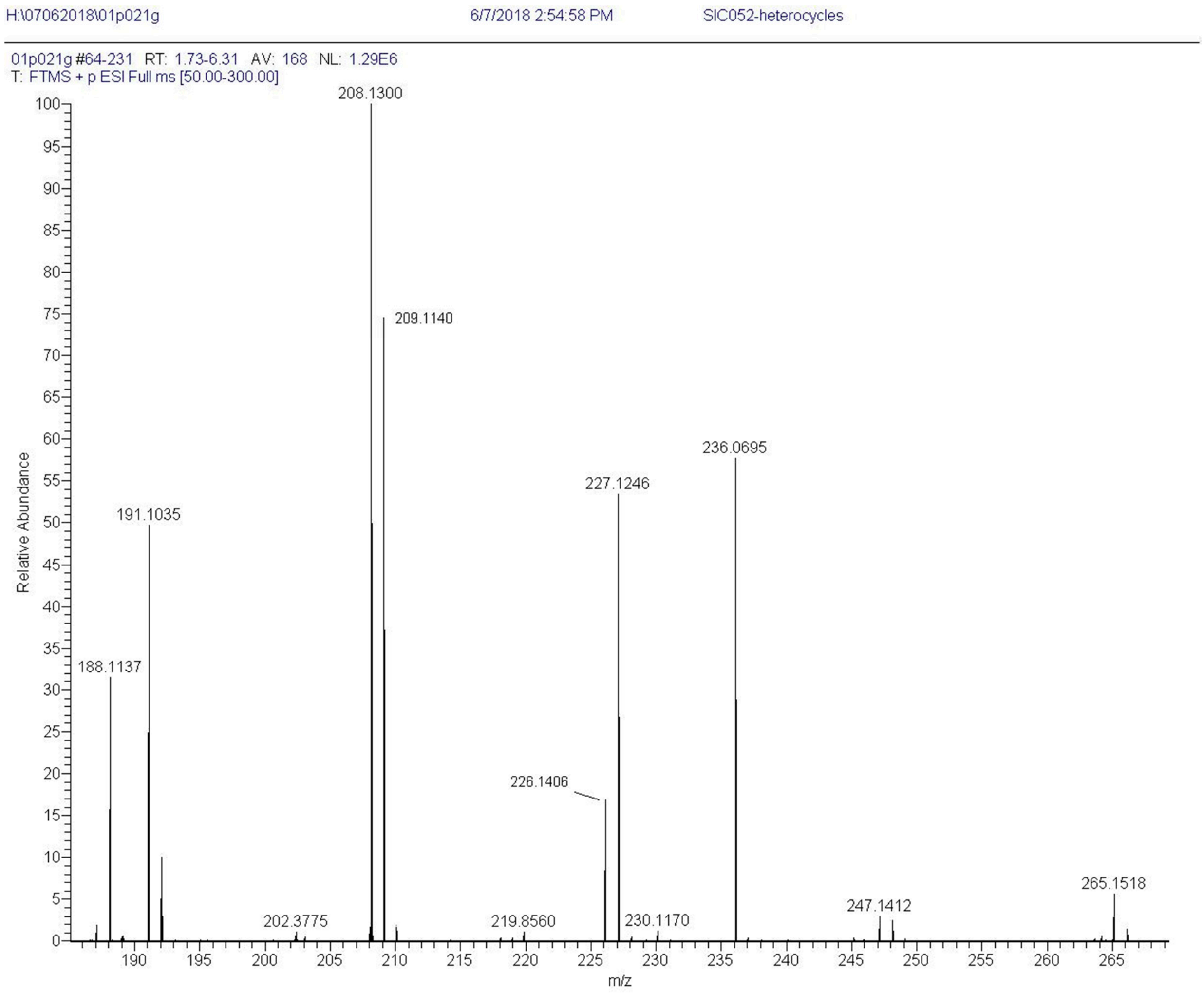

The above mass spectrum was obtained for a reaction run from 15 mg of cysteine and 114 mg of **1a**.HCl (10 eq.) in 1.5 mL of phosphate buffered water (pH 6.5).

Some mass attributions

Some of these attributions are obvious or at least probable. In other cases different structures are possible (and plausible). The presence of the "dimeric" and "trimeric" derivatives of **1a,** $(NCCH_2)_2NH$ and $(NCCH_2)_3N$, is possible. They are indicated in this list, as well as two possible $(NCCH_2)_2NH$ derivatives. However, in all our test reactions in which **1a** was heated alone at 45°C in water under slightly acidic conditions, $(NCCH_2)_2NH$ was formed only very slowly (2 percent after 100 days) and $(NCCH_2)_3N$ was never detected.

Blue: observed ; red: calculated for the given molecular formula.

57.0440, $C_2H_5N_2^+$ 57.0453, **1a** +H

75.0548, $C_2H_7N_2O^+$ 75.0558, **15**

76.0378, $C_2H_6NO_2^+$ 76.0388, GlyOH +H

96.0552, $C_4H_6N_3^+$ 96.0561, **19** or $(NCCH_2)_2NH$ +H

113.0817, $C_4H_9N_4^+$ 113.0827, **18**

114.0656, $C_4H_8N_3O^+$ 114.0667, **1h**

131.0923, $C_4H_{11}N_4O^+$ 131.0933

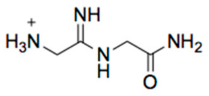

132.0762, $C_4H_{10}N_3O_2^+$ 132.0773, $GlyGlyNH_2$ +H

135.0660, $C_6H_7N_4^+$ 135.0670, $(NCCH_2)_3N$ +H or

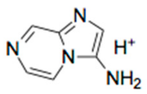 which would be obtained from 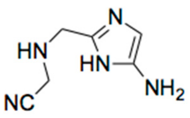

152.0926, $C_6H_{10}N_5^+$ 152.0936, $(NCCH_2)_2NC(=NH)CH_2NH_3$ or

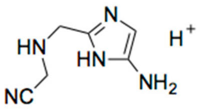 which would be obtained from **19** + **1**a

153.0766, $C_6H_9N_4O^+$ 153.0776, $(NCCH_2)_2NC(=O)CH_2NH_3$ +H or $NCCH_2NHCH_2C(=O)NHCH_2CN$ +H or

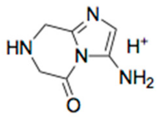 which would be obtained from 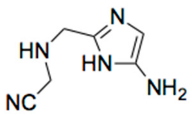

161.0375, $C_5H_9N_2O_2S^+$ 161.0385, **2a** +H

169.1193, $C_6H_{13}N_6^+$ 169.1202,

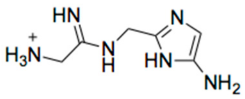

170.1032, $C_6H_{12}N_5O^+$ 170.1042, **21**

179.0481, $C_5H_{11}N_2O_3S^+$ 179.0490, Gly-Cys +H

208.1300, $C_8H_{14}N_7^+$ 208.1310, **20** +H

209.1140, $C_8H_{13}N_6O^+$ 209.1151,

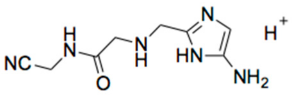

236.0695, $C_7H_{14}N_3O_4S^+$ 236.0705, **14** or Gly-Gly-Cys +H

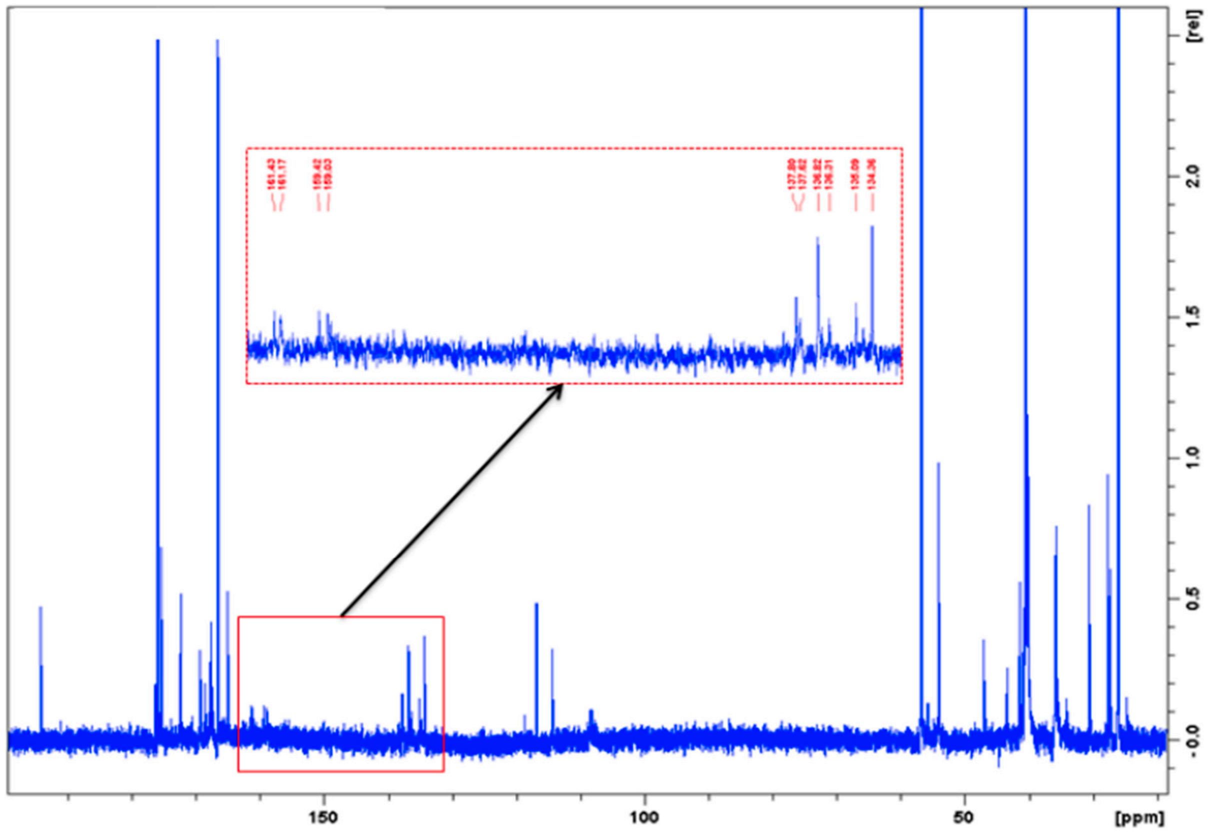

^{13}C NMR spectrum of a cysteine + excess **1a**.HCl reaction. The 5 biggest peaks correspond to GlyCys. The "imidazole zone" is enlarged, showing that more than two heterocyclic compounds were formed.

Theoretical calculations

From **1h**

1h + H_3O^+ + $2H_2O$ complex

B3LYP/6-31+g(d,p)scrf=(solvent=water)

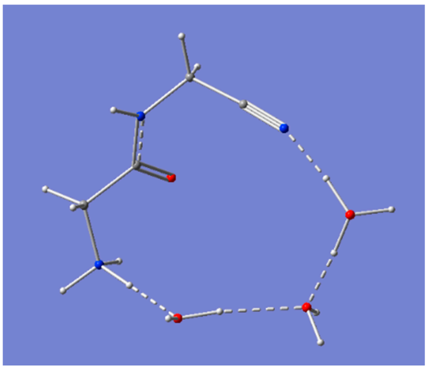

26

scf done: -626.376518

C	-0.615140	-2.184424	0.288736
N	0.493562	-2.406293	0.049415
C	-2.016773	-1.816401	0.575177
H	-2.191024	-1.941920	1.647252
H	-2.682194	-2.490736	0.033551
C	-1.740079	0.586400	0.860676
C	-2.015728	1.986041	0.314378
H	-2.011821	2.024464	-0.775171
H	-2.982281	2.339759	0.680258
N	-2.275332	-0.445951	0.168802
H	-2.715230	-0.289707	-0.729264
N	-0.944117	2.883055	0.831446
H	-0.032289	2.715553	0.327966
H	-1.197084	3.867742	0.728010
H	-0.788372	2.685309	1.826366
H	2.013361	-2.194661	-0.215196
O	2.999307	-1.945646	-0.403366
O	3.467422	0.444107	0.318266
H	4.283122	0.795352	-0.075638
H	3.571699	0.541427	1.279302
H	3.221116	-0.983781	-0.082192
H	3.601987	-2.593470	0.001057
O	1.431445	2.450797	-0.486696
H	2.046219	1.741261	-0.223246
H	1.408044	2.444748	-1.453643
O	-1.073920	0.437330	1.885560

TS from **1h**

B3LYP/6-31+g(d,p)scrf=(solvent=water)

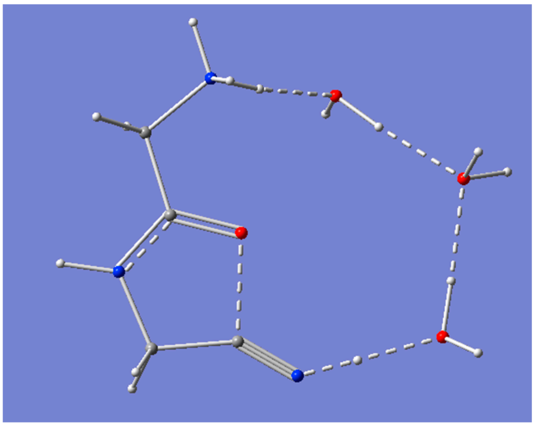

26

scf done: -626.355948

C	0.196128	-2.032414	0.093370
N	-0.920775	-2.426407	0.098282
C	1.632791	-2.416227	0.214441
H	1.893769	-3.067973	-0.625722
H	1.774351	-2.962495	1.151492
C	1.726590	-0.082457	0.008051
C	2.402094	1.266478	0.001715
H	2.654238	1.545508	1.028289
H	3.319103	1.242503	-0.588942
N	2.420857	-1.194794	0.193462
H	3.426188	-1.219688	0.310998
N	1.484538	2.300192	-0.551565
H	0.585202	2.404943	0.014278
H	1.954255	3.209869	-0.551632
H	1.227779	2.098182	-1.521957
H	-1.895042	-1.974147	0.009126
O	-3.267998	-1.296183	-0.029283
O	-3.144142	1.494962	-0.151215
H	-3.901499	1.864592	0.328725
H	-3.249578	1.797662	-1.066612
H	-3.241140	-0.314725	-0.101610
H	-3.866646	-1.613102	-0.720507
O	-0.792807	2.729400	0.838835
H	-1.598953	2.261801	0.530289
H	-0.750314	2.600442	1.796577
O	0.478061	-0.186364	-0.136048

Cyclized form.H^+ + 3 H_2O
B3LYP/6-31+g(d,p)scrf=(solvent=water)

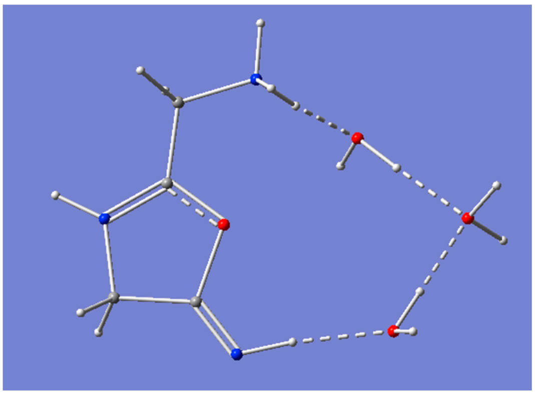

26
scf done: -626.361884

C	-0.672395	-1.839147	-0.103547
N	0.309993	-2.583696	-0.054256
C	-2.120101	-2.092011	-0.458930
H	-2.592685	-2.791024	0.235340
H	-2.226110	-2.458217	-1.482983
C	-1.804742	0.115950	0.043298
C	-2.046297	1.575091	0.275719
H	-2.361583	2.040497	-0.662007
H	-2.847710	1.696821	1.008050
N	-2.700906	-0.752412	-0.322153
H	-3.678869	-0.535230	-0.487058
N	-0.820121	2.262500	0.759535
H	-0.012872	2.241030	0.047345
H	-1.038842	3.247716	0.934957
H	-0.483974	1.874803	1.646060
H	1.211500	-2.147361	0.214927
O	2.900075	-1.522617	0.602139
O	3.552248	1.179121	-0.059731
H	4.263141	1.195263	-0.718591
H	3.894327	1.673897	0.700462
H	3.137204	-0.591773	0.417222
H	3.261161	-1.722889	1.475925
O	1.200309	2.346140	-0.976863
H	2.041989	1.922091	-0.691757
H	1.033663	2.056907	-1.884778
O	-0.607259	-0.382797	0.204581

From **16**

16 + H_3O^+ + 2 H_2O complex

B3LYP/6-31+g(d,p)scrf=(solvent=water)

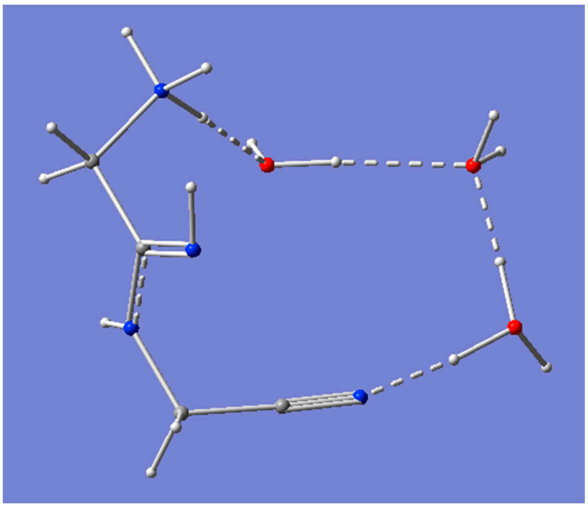

27

scf done: -606.484630

C	0.659821	2.106300	0.091956
N	1.720775	1.685600	-0.091513
C	-0.717528	2.576996	0.361376
H	-1.046490	3.168837	-0.497150
H	-0.695950	3.219389	1.244371
C	-2.025039	0.677256	-0.461466
C	-2.883139	-0.516953	-0.055452
H	-3.183039	-0.476482	0.991734
H	-3.779955	-0.560235	-0.674166
N	-1.621359	1.470581	0.583789
H	-1.697389	1.121215	1.528866
N	-2.125043	-1.800689	-0.250052
H	-1.245648	-1.842871	0.335756
H	-2.710969	-2.599082	0.009232
N	-1.686962	0.969736	-1.665897
H	-2.121915	0.349608	-2.347100
H	-1.855325	-1.936342	-1.227799
H	2.935070	0.767538	-0.277349
O	3.691965	0.067543	-0.422168
O	2.757102	-2.270357	-0.036595
H	3.306554	-2.841408	0.525026
H	2.638387	-2.750564	-0.872451
H	3.352252	-0.893207	-0.248322
H	4.462728	0.267543	0.136640
O	0.158991	-1.848231	1.273439
H	1.010257	-2.031368	0.832314
H	0.135260	-2.410694	2.060234

TS from **16** to **17**.H^+

B3LYP/6-31+g(d,p)scrf=(solvent=water)

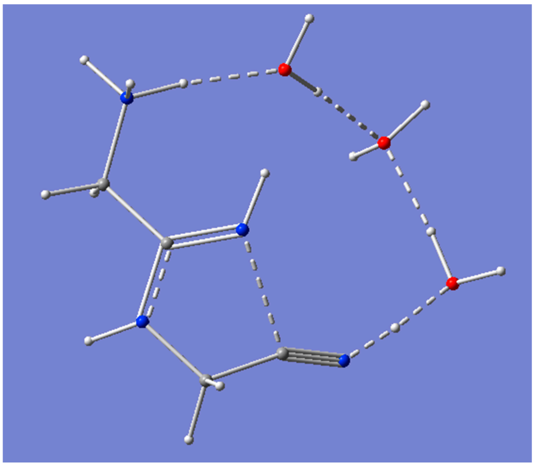

27

scf done: -606.472505

C	0.106392	-2.141800	0.135073
N	1.261684	-2.266429	0.024226
C	-1.328406	-2.536093	0.066154
H	-1.598930	-2.878799	1.072318
H	-1.433897	-3.365958	-0.635897
C	-1.670747	-0.181593	0.019205
C	-2.372568	1.005038	-0.614467
H	-2.033654	1.114927	-1.647265
H	-3.453761	0.861953	-0.616825
N	-2.133690	-1.407504	-0.353444
H	-2.775745	-1.523352	-1.124817
N	-2.076210	2.287242	0.094091
H	-1.046069	2.481735	0.166419
H	-2.505180	3.068991	-0.409236
N	-0.655704	-0.181836	0.808967
H	-0.211731	0.709833	1.023607
H	-2.464830	2.294525	1.041994
H	2.268700	-1.752540	0.083587
O	3.444332	-1.029067	0.066472
O	2.939188	1.579803	-0.719706
H	2.828021	1.593632	-1.683327
H	3.697015	2.158498	-0.541416
H	3.301021	-0.081847	-0.181154
H	3.933691	-1.041261	0.902784
O	0.632135	2.561738	0.600461
H	1.431325	2.286880	0.101039
H	0.904317	3.266313	1.204286

17 + 3 H_2O

B3LYP/6-31+g(d,p)scrf=(solvent=water)

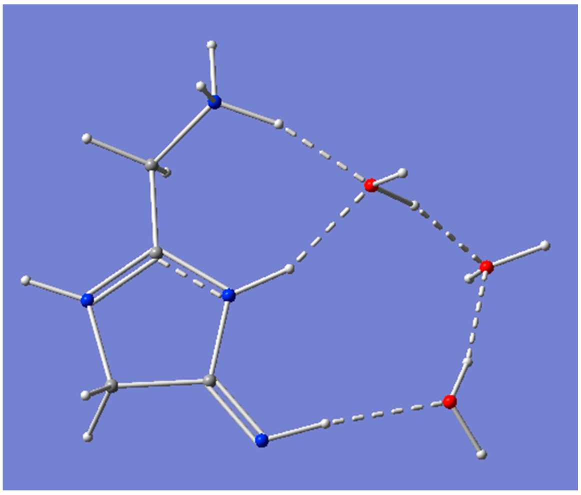

27
scf done: -606.521043

C	0.277484	-1.809809	-0.281470
N	-0.790667	-2.421615	-0.551159
C	1.645245	-2.427494	-0.021524
H	2.045219	-2.929301	-0.906626
H	1.621396	-3.130387	0.814715
C	1.736252	-0.147826	0.244074
C	2.273423	1.200080	0.637826
H	1.933210	1.415599	1.654275
H	3.361917	1.188274	0.629843
N	2.453347	-1.247414	0.311708
H	3.435220	-1.280461	0.557555
N	1.805479	2.315056	-0.243165
H	0.757262	2.368551	-0.308475
H	2.139508	3.207024	0.135410
N	0.476970	-0.402871	-0.114391
H	-0.232967	0.328238	-0.277375
H	2.180066	2.237613	-1.194042
O	-3.291375	-0.682023	-0.513197
O	-3.181087	1.838279	0.925703
H	-3.083431	1.788773	1.888334
H	-3.889059	2.479712	0.764443
H	-3.367466	0.126957	0.027074
H	-4.072606	-1.211483	-0.304915
O	-0.909397	1.984222	-0.568641
H	-1.699188	2.090346	0.015831
H	-1.209548	2.152055	-1.474161
H	-1.604754	-1.799151	-0.640679
